# National Cleft and Craniofacial Awareness and Prevention Month — July 2014

**Published:** 2014-07-04

July is National Cleft and Craniofacial Awareness and Prevention Month, an observance intended to raise awareness and improve understanding of birth defects of the head and face. Common craniofacial birth defects include orofacial clefts (cleft lip, cleft palate, or both), craniosynostosis (when the skull sutures join together prematurely), and anotia/microtia (when the ear is missing or malformed).

This year, CDC highlights research on the association between smoking during early pregnancy and orofacial clefts. Although the causes of most orofacial clefts are unknown, the 2014 Surgeon General’s report confirmed that maternal smoking during early pregnancy can cause orofacial clefts in babies (1). In the United States, approximately 7,000 babies are born with orofacial clefts each year (2). Many of those birth defects could be prevented if women did not smoke during early pregnancy.

Orofacial clefts occur very early in pregnancy. Health-care providers should encourage women who are thinking about becoming pregnant to quit smoking before pregnancy or as soon as they find out that they are pregnant. Additional information regarding National Cleft and Craniofacial Awareness and Prevention Month is available at http://www.nccapm.org/about.html.
